# Protein Oxidation in the Lungs of C57BL/6J Mice Following X-Irradiation

**DOI:** 10.3390/proteomes3030249

**Published:** 2015-08-19

**Authors:** Michal Barshishat-Kupper, Elizabeth A. McCart, James G. Freedy, Ashlee J. Tipton, Vitaly Nagy, Sung-Yop Kim, Michael R. Landauer, Gregory P. Mueller, Regina M. Day

**Affiliations:** 1 Department of Pharmacology, Uniformed Services University of the Health Sciences, Bethesda, MD 20814, USA; E-Mails: elizabeth.mccart@usuhs.edu (E.A.M.); atipton04@gmail.com (A.J.T.); 2 Department of Anatomy, Physiology and Genetics, Uniformed Services University of the Health Sciences, Bethesda, MD 20814, USA; E-Mails: james.freedy.ctr@usuhs.mil (J.G.F.); gregory.mueller@usuhs.edu (G.P.M.); 3 Operational Dosimetry Division, Armed Forces Radiobiology Research Institute, Uniformed Services University of the Health Sciences, Bethesda, MD 20889, USA; E-Mails: vitally.nagy@usuhs.edu (V.N.); sungyop.kim.ctr@usuhs.edu (S.-Y.K.); 4 Radiation Countermeasures Program, Scientific Research Department, Armed Forces Radiobiology Research Institute, Uniformed Services University of the Health Sciences, Bethesda, MD 20889, USA; E-Mail: michael.landauer@usuhs.edu

**Keywords:** thoracic irradiation, protein carbonylation, OxyBlot, mass spectrometry, 2-D gel electrophoresis, pulmonary fibrosis, radiation pneumonitis

## Abstract

Damage to normal lung tissue is a limiting factor when ionizing radiation is used in clinical applications. In addition, radiation pneumonitis and fibrosis are a major cause of mortality following accidental radiation exposure in humans. Although clinical symptoms may not develop for months after radiation exposure, immediate events induced by radiation are believed to generate molecular and cellular cascades that proceed during a clinical latent period. Oxidative damage to DNA is considered a primary cause of radiation injury to cells. DNA can be repaired by highly efficient mechanisms while repair of oxidized proteins is limited. Oxidized proteins are often destined for degradation. We examined protein oxidation following 17 Gy (0.6 Gy/min) thoracic X-irradiation in C57BL/6J mice. Seventeen Gy thoracic irradiation resulted in 100% mortality of mice within 127–189 days postirradiation. Necropsy findings indicated that pneumonitis and pulmonary fibrosis were the leading cause of mortality. We investigated the oxidation of lung proteins at 24 h postirradiation following 17 Gy thoracic irradiation using 2-D gel electrophoresis and OxyBlot for the detection of protein carbonylation. Seven carbonylated proteins were identified using mass spectrometry: serum albumin, selenium binding protein-1, alpha antitrypsin, cytoplasmic actin-1, carbonic anhydrase-2, peroxiredoxin-6, and apolipoprotein A1. The carbonylation status of carbonic anhydrase-2, selenium binding protein, and peroxiredoxin-6 was higher in control lung tissue. Apolipoprotein A1 and serum albumin carbonylation were increased following X-irradiation, as confirmed by OxyBlot immunoprecipitation and Western blotting. Our findings indicate that the profile of specific protein oxidation in the lung is altered following radiation exposure.

## 1. Introduction

Pulmonary injuries are limiting factors for the use of radiotherapy for the treatment of thoracic cancers [1,2,3,4,5,6]. Clinically significant lung injury has been estimated to occur in ~10%–30% of patients undergoing radiotherapy for cancers in the thoracic region [7,8], and pulmonary effects have also been observed in patients undergoing radiotherapy for cancer treatment or bone marrow transplantation [9,10,11,12]. Lung injury is also of medical concern following accidental exposures to ionizing radiation, with delayed lung toxicity a significant cause of morbidity and mortality in patients who survive acute illness following nuclear accidents [2,13,14]. Two distinct phases of lung injury have been recognized following radiation exposure. Radiation pneumonitis typically manifests within several weeks to several months following exposure and is characterized by low-grade fever, mild dyspnea, congestion, and an unproductive cough [2,15]. Radiation-induced lung fibrosis can develop within a month to a year after radiation exposure, and is characterized by diffuse lung remodeling that can progress for up to five to six years, resulting in decreased function lung volume [2,15]. While well-characterized clinically, there are currently no accepted approaches for prevention or treatment of radiation pneumonitis or fibrosis, and mortality is as high as 50% for each phase following accidental radiation exposure [16].

The sensitivity of the lung to radiation is not completely understood. It is hypothesized that the lung’s high oxygen content compared with other organs renders it more sensitive to radiation-induced damage due to the generation of reactive oxygen species (ROS) [17]. Primary radiation injury inflicts direct damage to the biological components of cells, particularly in rapidly dividing cells, but secondary effects can arise from the generation of destructive free radicals that initiate and propagate chain reactions [17,18]. The delayed onset of radiation-induced lung injury is believed to be the result of a multi-step process involving repeated episodes of inflammation followed by failed repair due to loss of regenerative properties of the normal tissue and their replacement with activated fibroblasts [17,19].

DNA damage, including single- and double-strand breaks, has long been considered the most critical biological effect of ionizing radiation and ROS, inducing both cell death and mutations that can result in tumorigenic transformation [20]. However, protein damage by radiation has been demonstrated to have a significant impact on cellular viability and clonogenicity [21,22,23]. Using primary lung endothelial cell cultures, we previously demonstrated that protein oxidation by radiation can lead to endoplasmic reticulum (ER) stress, that can lead to programmed cell death and accelerated senescence within 24 h postirradiation [23]. Other studies demonstrated that protein expression can be significantly altered following radiation exposure [24].

We previously provided evidence that proteins in the liver and bone marrow of mice are oxidized in normal tissue and that the profile of protein oxidation is altered in response to ionizing radiation exposure, with some proteins displaying enhanced carbonylation and other proteins displaying reduced carbonylation at 24 h postirradiation [25,26]. We found that the overall pattern of protein oxidation differed between liver and bone marrow. Here we have investigated the alterations in protein oxidation induced at 24 h following irradiation of the lung using two-dimensional (2-D) gel electrophoresis and OxyBlot detection of protein carbonylation. Peptide mass fingerprinting was used for the identification of carbonylated proteins. The present findings show that radiation exposure alters the profile of protein carbonylation in the lung, and that there are both similarities and differences in the identified proteins that are carbonylated in the lung compared with other tissues.

## 2. Experimental Section

### 2.1. Chemicals

Except where noted, chemicals were purchased from Sigma-Aldrich (St. Louis, MO, USA).

### 2.2. Animals

C57BL/6J female mice (The Jackson Laboratory, Bar Harbor, ME, USA) weighing 17.5–21.5 g were irradiated at 12–14 weeks of age. Mice were maintained in a barrier facility for animals accredited by the Association for Assessment and Accreditation of Laboratory Animal Care International. Mice were housed in groups of four per cage. Animal rooms were maintained at 21 ± 2 °C, 50% ± 10% humidity, and 12-h light/dark cycle with commercial freely available rodent ration (Harlan Teklad Rodent Diet 8604, Frederick, MD, USA). Acidified water (pH = 2.5–3.0) was available *ad libitum* to control opportunistic infections [27]. All procedures with animals were performed in compliance with guidelines from the National Research Council for the ethical handling of laboratory animals, and were approved by the Institutional Animal Care and Use Committee of the Armed Forces Radiobiology Research Institute (AFRRI, Bethesda, MD, USA).

### 2.3. Thoracic Irradiation

For thoracic irradiation, mice (12–14 weeks of age) were anesthetized with intraperitoneal injections of 150 mg/kg ketamine plus 18 mg/kg xylazine. Anesthetized mice were irradiated in the prone position in Lucite jigs (3 mm thick) (Precision Machine and Tool, Beltsville, MD, USA) to prevent movement, and forelimbs were secured out of the radiation field. Thoracic X-ray images were taken to confirm thoracic positioning in the radiation field. The jigs held animals in the weight range of 17–22 g without constriction of the thorax. Following irradiation, anesthetized animals recovered on warming pads prior to being returned to their original cages.

The Philips Industrial X-ray Machine (Royal Philips, Amsterdam, The Netherlands) at the Armed Forces Radiobiology Research Institute (AFRRI) supplied an uncollimated radiation field diameter of a ~30 cm at 50 cm source to surface distance (SSD). Although the field of the irradiator was fairly non-uniform, its polar symmetry allowed the identification of four thorax-sized areas with central axis dose rates identical within ± 1.2%. To use them, a custom 9 mm thick lead shield (42.6 × 27.9 cm^2^) with four openings (3.18 cm × 2.22 cm) was fabricated (Precision Machine and Tool, Beltsville, MD, USA). The exposure rate variations over each thoracic area were within ±2.5%. Irradiation was performed at 250 kVp and 12.0 mA filament current. The inherent beryllium filter was used with 1.25 mm Cu and 0.95 mm Al filters for beam hardening. The half-value layer (HVL) of the resulted beam was determined according to the American Association for Physicists in Medicine Task Group-61 protocol to be 2.3 mm Cu [28]. The exposure dosage was calibrated using Lucite cylindrical phantoms (2.54 cm diameter × 7.62 cm length) located under the holes of the shield, each containing in its core three alanine dosimeters (FarWest Technologies, San Diego, CA, USA). The filtering necessary for the beam hardening decreased the dose rate at the mouse core to ~0.5 Gy/min. Doses to the alanine dosimeters were measured with an e-Scan electron paramagnetic resonance (EPR) spectrometer (Bruker Biospin, Billerica, MA, USA) [29]. The reproducibility of signals of replicate dosimeters was ≥ 0.5%. The EPR spectrometer at AFRRI was calibrated with standard alanine dosimeters purchased from the National Institute of Standards and Technology (Gaithersburg, MD, USA), directly traceable to national radiation standards. An additional verification of the calibration accuracy was performed by an intercomparison with the British National Physics Laboratory (Teddington, Middlesex, UK). Animal exposures were based on the charge measured with a monitor ionization chamber Exradin A12 (Standard imaging, Middleton, WI, USA) permanently fixed in the radiation field away from the apertures, which was cross-calibrated with the dose to alanine. This technique made it possible to eliminate possible inaccuracies due to current fluctuations in the filament during the long irradiation times.

### 2.4. Protein Oxidation Detection by OxyBlot

Mice were euthanized and lungs were perfused by injection of phosphate buffered saline (PBS) through the left aorta. Protein was extracted from the left lungs using 1 mL of the following protein lysis buffer (PLYB): 10 mM Na_4_P_2_O_7_, 50 mM NaF , 50 mM NaCl, 1 mM EDTA (pH = 8.0), 50 mM HEPES, 1% Triton X100, adjusted to pH = 7.5. To this buffer 2 mM Na_3_VO_4_, 1 mM PMSF, 1 mM DTPA, and cOmplete Mini Protease Inhibitor tablet (1 tablet/10 mL buffer; Roche Applied Science, Indianapolis, IN, USA). The OxyBlot Oxidized Protein Detection Kit was purchased from Chemicon (Millipore, Billerica, MA, USA) and performed as used as previously described [26]. 2,4-dinitrophenylhydrazine (DNPH) derivatization was performed for 15 min at room temperature following manufacturer’s instruction using 10 µg of protein. After DNPH derivatization, proteins were subjected to two-dimensional gel electrophoresis (see below). For immunodetection, proteins were transferred to polyvinylidene difluoride (PVDF) membranes. Membranes were blocked with 1% BSA in phosphate buffer saline (PBS) containing (*w*/*v*) 0.05% Tween-20 for 1 h at room temperature. After overnight 4 °C incubation with anti-DNP antibody, blots were washed three times and secondary rabbit antibody was added for 1 h, ambient temperature. Blots were washed and developed using a SuperSignal West Pico chemiluminescence detection system (Thermo Scientific, Rockford, IL, USA).

### 2.5. Two Dimensional (2-D) Gel Electrophoresis

Protein extraction from the lung was performed as previously described for the bone marrow [26]. Briefly, 4 volumes of 10 mM DNPH (in 2 M HCl) were added to 200 μg protein extract of each sample and incubated for 30 min at room temperature. A final concentration of 15% of ice-cold trichloroacetic acid (TCA) was added to each sample and incubated for 10 min on ice. Samples were centrifuged for 10 min at 16,000× *g* at 4 °C. Pellets were washed three times with ethanol ethyl acetate (1:1) and centrifuged at 16,000× *g* for 15 min, 4 °C. Two-dimensional gel electrophoresis was performed according to manufacturer’s instructions (2-D Starter Kit, Bio-Rad Laboratories, Hercules, CA, USA). Pellets were resuspended in 2-D rehydration buffer. Protean Isoelectric Focusing (IEF) Cell (Bio-Rad Laboratories) was used for first dimension separation. Samples were then applied to immobilized pH gradient strips (nonlinear pH 5–8) for 1 h at ambient temperature and then subjected to IEF. Protein IEF strips were reduced and alkylated by incubating for 10 min each in Equil Buffers 1 and 2 according to the manufacturer’s instructions. The strips were embedded in 0.7% agarose on top of 4%–20% acrylamide gels (Criterion precast gels, Bio-Rad Laboratories) for second dimension electrophoresis. Proteins were transferred to PVDF membranes using a shortened protocol (20 min, 20 V) so that proteins remaining in the partially transferred gels could be visualized by Coomassie staining (SimplyBlue Safe Stain, Invitrogen, Carlsbad, CA, USA). Carbonylated proteins detected by OxyBlot were mapped to corresponding features on Commassie stained gels (Bio-Rad).

### 2.6. Peptide Mass Fingerprinting for Protein Identification

Protein identifications were assigned based on peptide mass fingerprinting performed [26,30]. Excised protein spots were destained, then equilibrated with 0.2 mL of 100 mM NH_4_HCO_3_/50% acetonitrile for 45 min at 37 °C, dehydrated in 100 μL 100% acetonitrile, and dried under vacuum. Gel pieces were rehydrated overnight at 37 °C in 40 mM NH_4_HCO_3_/10% acetonitrile containing 20 ng/μL trypsin (Trypsin Gold, Mass Spectrometry Grade, Promega, Madison, WI, USA). Peptides were recovered in sequential (60 min, room temperature) extractions with 1.0% trifluoroacetic acid (TFA, 75 µL) followed by two rinses with 50% acetonitrile/5% TFA (50 µL each). The collections were pooled, dried under vacuum and dissolved in 1% TFA (10 µL). The peptides were then purified and concentrated using a C18 Zip Tip^®^ (Millipore Corporation, Billerica, MA, USA) and mixed with alpha-cyanohydroxycinnamic acid matrix (10 mg/mL in 50% acetonitrile/0.1% TFA) containing bradykinin (1060.5692 daltons; 50 fmol/mL) and adrenocorticotropic hormone fragment 18–39 (2465.1989 daltons; 150 fmol/mL; AnaSpec, San Jose, CA, USA) as internal standards. Samples were analyzed by matrix-assisted laser desorption ionization time-of-flight (MALDI-TOF) mass spectrometry using a Voyager MALDI-TOF DE-STR instrument (PE Biosystems, Framingham, MA, USA). The mass spectrometer was operated in reflectron mode with an accelerating voltage of 20,000 V, a grid voltage of 76.13% and a guidewire voltage of 0.003%. Peptide mass data were used to query the National Center for Biotechnology Information (NCBI; Bethesda, MD, USA) protein sequence database accessed through the ProteinProspector MS-Fit search engine [31,32]. Protein assignments were based on: (1) probability scores derived from the Molecular Weight Search (MOWSE) of ProteinProsector software (The Regents of the University of California, Oakland, CA, USA, v 5.14.2), based on mass matches and percent protein sequence coverage, and (2) minimal frequency of four observations across five separate experiments. Published evidence supporting the assignments was also taken into account.

### 2.7. DNP-Labeled Protein Confirmation by Immunopercipitation and Western Blotting

Fifty micrograms of lung proteins were labeled with DNPH as described before for the two dimensional electrophoresis [33]. Samples were then pre-cleared by adding 10 μL of protein A/G PLUS-Agarose (A/G-agarose; Santa Cruz Biotechnology, Santa Cruz, CA, USA) and incubated at 4 °C for 2 h on a tube rotator. After centrifugation, to remove the A/G-agarose, the supernatant was transferred to a new tube and 10 μL of ant-DNP antibody was added and incubated 4 °C on a tube rotator overnight. A/G-agarose (20 μL) was added and the samples were rotated for an additional 2 h. The samples were centrifuge at 16,000 × *g* for 2 min and washed twice with PLYB buffer before adding 30 μL of Laemmli buffer (Bio-Rad) containing β-mercaptoethanol. Samples were boiled for 5 min at 95 °C and SDSPAGE was performed. Proteins were transferred to PVDF membrane and were detected with the following antibodies (1:1000, albumin and apolipoprotein A1; Santa Cruz Biotechnology). Band densitometry was performed on samples on the same gel. Wright Cell Imaging Facility ImageJ software (National Institutes of Health, Bethesda, MD, USA, version 1.48j) was used for densitometry analysis [34]. Control Western blots for each antibody was performed on lysates prior to DNP modification to ensure that equivalent amounts of lysate were used in each immunoprecipitation and to allow normalization between samples.

### 2.8. Statistics

The Kaplan-Meier method was used to plot survival as a function of time, and comparisons of the survival curves were analyzed by the log rank test. The mean survival time (MST) was determined by calculating the average time to death within 210 days; survivors were assigned a value of 210. Significant differences between the two groups for Western blots were statistically determined by the Student’s *t* test.

## 3. Results and Discussion

### 3.1. Survival Studies: 17 Gy Thoracic Irradiation Induces Lung Fibrosis within 180 Days

**Figure 1:**
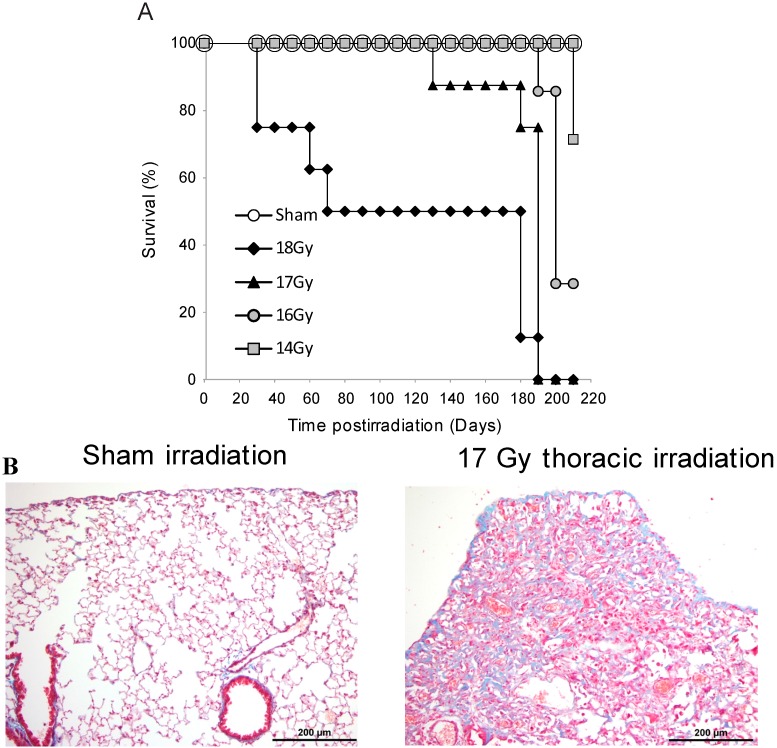
Seventeen Gy thoracic irradiation is sufficient to induce pulmonary fibrotic remodeling within 200 days (**A**) Kaplan-Meier plot of survival following 14, 16, 17, or 18 Gy of thoracic irradiation (*N* = 7–8); (**B**) In a separate experiment, lung tissue was obtained from sham-irradiated and irradiated mice exposed to 17 Gy thoracic irradiation, at 180 days postirradiation. Tissues were fixed, paraffin embedded, and used for Masson’s Trichrome stain. Collagen appears as blue. Representative data are shown (N = 6).

We wished to identify proteins oxidized in the lung at 24 h following a level of radiation exposure that would be sufficient to induce delayed pulmonary fibrosis, as these oxidized proteins could contribute to early alterations in cell function and survival. We first investigated the dose of radiation required to induce mortality within ~180 days in C57BL/6J mice using the Philips Industrial X-ray Machine. C57BL/6J mice received 14, 16, 17 or 18 Gy (0.6 Gy/min) thoracic irradiation, using a lead shield to isolate exposure to the thoracic region. This time of mortality was previously correlated with lung fibrosis in C57BL/6 mice using other radiation sources [35]. Survival was monitored up to 210 days postirradiation (Figure 1A). The percent survival and MST at 210 days postirradiation for mice that received 14 Gy thoracic irradiation was 71% (MST = 209 days), 16 Gy was 29% (MST = 196 days), 17 Gy was 0% (MST = 178 days), and 18 Gy was 0% survival (MST = 109 days). Thoracic exposure (18 Gy) resulted in some early time points of mortality (e.g., days 9, 12, and 58 postirradiation), suggesting that injuries to organs in addition to the lung had occurred. This treatment group (18 Gy) exhibited mortality significantly (*p* < 0.05) earlier than the other irradiated groups. Mice in the 14 and 16 Gy groups survived significantly (*p* < 0.05) longer than animals in the 17 Gy group. To confirm that pulmonary remodeling occurred, in a separate group of mice, examination of lung tissue of surviving mice at 180 days following 17 Gy provided evidence of extensive collagen deposition, characteristic of fibrotic remodeling (Figure 1B).

### 3.2. Radiation-Induced Protein Carbonylation in Lung Tissue

We previously investigated radiation-induced alterations in protein oxidation in liver and bone marrow tissues [25,26]. These two studies provided evidence that the identities of protein carbonylation are tissue specific in control tissue, and demonstrated that radiation-induced alterations in carbonylation in a fixed set of proteins was also tissue specific. Because the lung is highly susceptible to oxidative stress, including ionizing radiation, we investigated the alterations in protein carbonylation at the dose of radiation that we determined was sufficient to induce pulmonary fibrotic remodeling.

**Figure 2:**
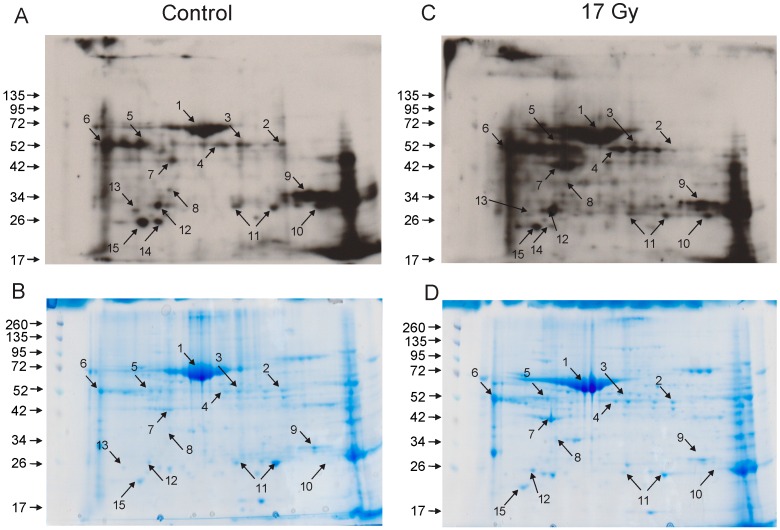
Two dimensional gel electrophoresis and OxyBlot analysis of sham-irradiated and irradiated lung proteins. C57BL/6J mice were either sham irradiated (control, **A**,**B**) or exposed to 17 Gy thoracic irradiation (17 Gy, **C**,**D**). Lung cell lysates were obtained 24 h postirradiation and protein lysates from individual mice for used for one gel. Upper panels: OxyBlots (**A**,**C**). Lower panels: Coomassie stained gels (**B**,**D**). Proteins detected by OxyBlot are indicated with numbered arrows. OxyBlots and gels are representative of N = 4 gels per group.

Two dimensional gel electrophoresis and OxyBlot analysis allowed the detection of 15 carbonylated proteins in sham-irradiated (control) and irradiated lung samples (17 Gy thoracic irradiation, 0.6 Gy/min) (Figure 2). All 15 were present in both control and irradiated lung samples. Radiation exposure altered the pattern of protein carbonylation, increasing the apparent carbonylation of 5 of the proteins (spots 1, 4, 5, 7, and 12). However, radiation had no effect on the carbonylation status of four of the proteins (spots 3, 6, 8, and 11), and radiation exposure resulted in relative decrease in the carbonylation of six proteins (spots 2, 9, 10, 13, 14, and 15). It is important to note that radiation exposure did not induce a general carbonylation of all lung proteins, but instead resulted in oxidation of a specific subset of proteins, suggesting that an ordered process results in oxidation of proteins with specific structural features.

### 3.3. Identification of Carbonylated Proteins from Control and Irradiated Lung Tissue

Carbonylated proteins were excised from corresponding Coomassie-stained gels and subjected to peptide mass fingerprinting for protein identification. Definitive identifications were obtained only seven of the fifteen proteins, listed in Table 1 and Table S1 (Protein Prospector MS Fit data). For some features, more than one protein was identified. For example, the feature identified as apolipoprotein A1, also produced an alternative an alternative identification of metastasis-associated protein MTA1, but the MOWSE score for the alternative identification was 7.68 × 10^5^ compared with 1.79 × 10^7^ for apolipoprotein A1. For selenium binding protein, β-tubulin 2D chain was also identified, but not in all samples (3 of 5).

**Table 1:** Carbonylated proteins were identified by peptide mass finger printing. Numbers correspond to spot numbers identified in 2-D gels of control and irradiated lung proteins (Figure 2).

Spot No.	Protein ID	Number of Peptides	% Coverage
1	Serum albumin	20	40
2	Selenium binding protein-1 (SBP1)	12	36
6	Alpha-1-antitrypsin	9	26
7	Actin, cytoplasmic	9	36
9	Carbonic anhydrase 2 (CAII)	9	50
11	Peroxiredoxin-6	16	75
12	Apolipoprotein A1	15	43

**Figure 3:**
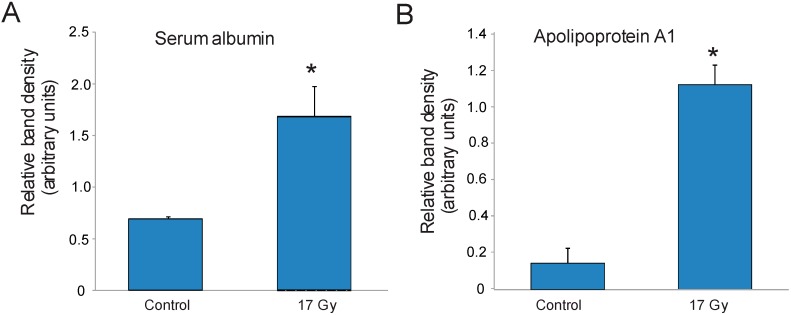
Confirmation of carbonylation on serum albumin and apolipoprotein A1. C57BL/6J mice were sham irradiated (control) or exposed to 17 Gy thoracic irradiation. Lung cell lysates were obtained 24 h postirradiation from three animals per group and DNP-labeled for immunoprecipitation with the OxyBlot antibody. Immunoprecipitated proteins were then Western blotted for (**A**) serum albumin or (**B**) apolipoprotein A1. Bar indicates average of band densitometry of three animals per condition; * indicates *p* < 0.05.

To confirm the identifications of the seven proteins listed in Table 1, we performed immunoprecipitation for DNP labeling followed by Western blotting for the target proteins. We found that DNP labeling hampered the binding of some antibodies to their target proteins in Western blotting. We confirmed the carbonylation of serum albumin and apolipoprotein A1, both of which had increased carbonylation in protein extracted from lung tissue following radiation exposure compared with control lung tissue (Figure 3). The other five proteins could not be confirmed using this technique. Control Westerns were performed on lysates to confirm that these protein levels did not significantly differ with radiation (data not shown).

Findings from our laboratory and others demonstrate that oxidative stress from chemical toxin exposure, radiation exposure, or due to inflammatory disease states can increased oxidation of proteins that function as enzymes as well as other proteins [24,25,26,36,37,38,39]. Our current findings indicate that there are both similarities and differences between the oxidized proteins in the lung in response to radiation and in other tissues.

Serum albumin has been previously identified as a target of radiation-induced protein oxidation in a number of studies including an *in vitro* study of protein oxidation [40] and our previous report on carbonylated proteins in the liver in sham and 7.5 Gy total body irradiated animals [25]. Serum albumin has also been demonstrated to be carbonylated in other conditions of oxidative stress, including in response to cigarette smoke [41,42], ischemia [43], uremic atherosclerosis [44], and chronic kidney disease [45]. Additionally, serum albumin was also observed to have increased carbonylation in rats fed high-fat, high-sucrose diets [46,47]. Carbonylation of serum albumin in the bronchoalveolar lavage fluid was observed in patients with asthma [48]. Because serum albumin has been demonstrated to bind and detoxify endotoxin in healthy individuals [49], it is possible that this protein may also function as a scavenger and/or antioxidant under some circumstances. The function of serum albumin carbonylation is not known, although it is known that oxidation of the protein likely modifies its antioxidant properties and its transport functions [42].

Selenium binding protein-1 (SBP1) is a member of the class of selenium binding proteins that covalently binds selenium but for which the physiological function remains largely unknown [50]. SBP1 has been demonstrated to play a crucial role in controlling the oxidation/reduction and redox homeostasis in a number of physiological processes [51]. A number of studies have demonstrated that SBP1 expression is reduced in a variety of cancers, and that reduced expression is correlated with poor prognosis in cancer patients [52]. SBP1 was identified by our laboratory as a protein that is carbonylated in sham-irradiated liver tissue but has reduced carbonylation following radiation exposure [25], in agreement with our current findings in lung tissue. The function of carbonylation of SBP1 is not known.

Alpha-1-antitrypsin is a member of the serpin protease inhibitor family with diverse physiological functions including modulation of inflammation and extracellular matrix remodeling [53]. Alpha-1-antitrypsin has been demonstrated to be one of two primary carbonylated proteins in the plasma of patients with chronic heart failure [54]. Alpha-1 antitrypsin is oxidized in other conditions of inflammation or oxidative stress including in the bronchoalveolar lavage fluid of asthma patients [48] and in the plasma and cerebral spinal fluid of Alzheimer’s patients [55,56]. Our laboratory previously demonstrated a reduction of carbonylated alpha-1 antitrypsin in the liver of mice exposed to 8.5 Gy total body irradiation compared with sham-irradiated animals [25].

Cytoplasmic actin is a ubiquitous protein present in all cell types, and was demonstrated to be the target of oxidation following 1,3-dinitrobenzene exposure and in HL-60 leukemia cells following peroxide exposure [36,57]. Carbonylation of actin in skeletal muscle has been reported in a number of animal models of muscle dysfunction [58]. Our laboratory also identified actin carbonylation in the bone marrow at 24 h after 7.5 Gy total body irradiation in mice [26]. We also observed decreased carbonylation of cytoplasmic actin in liver tissue following exposure to 8.5 Gy total body irradiation in mice [25].

Carbonic anhydrase 2 (CAII) is a regulator of acid-base homeostasis, and like actin was previously shown to be carbonylated in skeletal muscle after exposure to high levels of reactive oxygen species as well as other models of muscle dysfunction [58]. Our laboratory also identified carbonic anhydrase as a target of radiation-induced protein oxidation in the bone marrow, at 24 h after 7.5 Gy total body irradiation in mice [26].

Peroxiredoxins are a family of antioxidant enzymes that are ubiquitously expressed in mammalian cells in which a redox-active cysteine is responsible for detoxification of peroxide [59]. Peroxiredoxin-6 was demonstrated to be carbonylated in response to endothelin-1 receptor signal transduction cascades in cultures of pulmonary artery smooth muscle cells [33,60]. Peroxiredoxins were also found to be heavily carbonylated in the kidney of aging mole rats [61], and in the temporal cortex of aged rats in a model of Alzheimer’s disease [62]. The effect of carbonylation of peroxiredoxin activity has not been determined, but recent data indicates that peroxiredoxin can be decarbonylated via a thiol-dependent mechanism that likely involves glutatredoxin-1 enzyme [60].

Apolipoprotein A1 is the major protein component of the high-density lipoprotein complex in plasma and plays a crucial role in lipid transport and metabolism [63]. Apolipoprotein carbonylation has been observed in the cerebral spinal fluid of Alzheimer’s patients [56], and in the plasma of patients undergoing chemotherapy for cancer treatment [64]. Increased levels of carbonylated apolipoprotein A1 was identified in the amniotic fluid of women carrying Down syndrome fetuses, providing evidence that oxidative stress is an early characteristic of Down syndrome [65]. Oxidized apolipoprotein A1 was also observed in the serum of rats on a high fat diet [66], and in the plasma of type 2 diabetic patients or rheumatoid arthritis [67,68,69]. It was noted that carbonylated apolipoprotein A1 enhanced macrophage release of tumor necrosis factor-α (TNF-α), suggesting that this protein modification could contribute to TNF-α-mediated toxicity [64]. There are indications that some oxidative modification of apolipoprotein A1 result in decreased antioxidant capacity of the protein and/or decreased cholesterol acceptor activity [70,71,72], but it remains unknown whether either of these functions are affected by carbonylation.

## 4. Conclusions

Here we have investigated specific protein carbonylation in sham irradiated and 17 Gy thoracic irradiated lungs. Our findings indicate that 17 Gy is a dosage of irradiation required for the induction of irreversible pulmonary fibrosis in C57BL/6J mice at ~120 days. Although the overall pattern of protein carbonylation in the lungs of normal mice differs from those observed in liver and bone marrow, there are some proteins oxidized in common with other organ systems. Most importantly the effect of irradiation on the overall status of protein carbonylation is remarkably specific, affecting only a small subset of the entire cellular proteome. The specificity of protein oxidation may provide a clinically useful fingerprint for protein modification following radiation exposure. Future studies may be directed toward determining whether early protein carbonylation is linked to delayed lung fibrosis, which occurs ~180 days postirradiation in this model.

## Supplementary Materials

Supplementary File 1
